# The Effects For Growth of Perioperative Enteral Feeding in Neonates With Tga

**DOI:** 10.1186/2197-425X-3-S1-A495

**Published:** 2015-10-01

**Authors:** H Ebishima, K Kurosaki, I Shiraishi

**Affiliations:** National Cerebral and Cardiovascular Center, Pediatric Cardiology, Suita, Japan

## Introduction

Although the physical and nutritional benefits of early enteral feeding in neonates are well-known, it has not yet to be controversial that early enteral feeding in preoperative term neonates with unstable hemodynamics due to congenital heart diseases is safe or risky.

## Objectives

This retrospective study was designed to assess the effects for growth of enteral feeding in neonates with transposition of the great arteries (TGA) awaiting surgical repair.

## Methods

We reviewed records of 25 neonates, less than 28 days of age, with TGA undergoing surgical repair between January 2011 and December 2014.

## Results

They were 18 enteral feeding recipients and 7 non-recipients in preoperative term. Among 2 groups, there were no differences in sex, gestational age, body weight at birth, or day of age at the operation. Multivariable analysis was performed to evaluate preoperative calorie intake, postoperative length of stay, durations of ventilation and vasopressor use and body weight gain. There were no differences in postoperative length of stay or durations of ventilation and vasopressor use. Enteral feeding recipients were associated with high calorie intake in preoperative term and large body weight gain after operation, especially during 6 months after operation, large weight increase per day (least square mean difference, 6 gram per day, p = 0.04). No patients had necrotizing enterocolitis.

## Conclusions

Because this is a small retrospective review, conclusions regarding the safety of enteral feeding in TGA neonates are speculative. However, in this analysis of infants with TGA undergoing surgery, preoperative enteral feedings was well tolerated and beneficial to the growth.

